# Uncovering the Framework of Brain-Mind-Body in Creative Insight

**DOI:** 10.3389/fpsyg.2016.01327

**Published:** 2016-08-31

**Authors:** Wangbing Shen, Yuan Yuan, Chang Liu, Jing Luo

**Affiliations:** ^1^Institute of Applied Psychology and School of Public Administration, Hohai UniversityNanjing, China; ^2^School of Psychology and Lab of Cognitive Neuroscience, Nanjing Normal UniversityNanjing, China; ^3^School of Rehabilitation Science, Nanjing Normal University of Special EducationNanjing, China; ^4^Beijing Key Laboratory of Learning and Cognition, Capital Normal UniversityBeijing, China

**Keywords:** creative insight, embodied cognition, brain-mind, mind-body, meditation

Creative insight (CI) has been extensively investigated in psychology and cognitive neuroscience, which is defined as the process in which impasses the solver encountered are broken by restructuring and then leads to a peculiar experience accompanying the sudden solution. Numerous studies have greatly extended our knowledge concerning neurocognitive mechanism of CI, and offered robust supports for brain-mind principle of CI (Sternberg and Davidson, 1995). With the emergence of embodied approach (Gallagher, 2015), the relationship between body and CI is a burgeoning issue that has been widely concerned. Here, we elaborated the theoretical framework of triadic interaction of brain-mind-body (TIBMB) for CI, which would deepen our understandings on mind-body relationship and the essence of CI.

## The interaction of brain-mind-body: the fundamental idea and supportive evidence

The TIBMB is rooted in the brain-mind principle in psychology and can be traced back to the central issue of the relation between mind and body in philosophy (Gallagher, 2015). This theory argues that CI can be achieved by the TIBMB and that CI also conversely exerts critical influences on these three agents. Interfering with one of the three subsystems may also partly change the CI process.

The term “mind” *commonly* refers to a collection of mental processes rather than a substance or spirit (Fischbach, 1992), similar to the elements that are manifested when understanding a joke, solving a difficult problem, or experiencing happiness. Of note, rather than being the process of CI itself, the mind *here* refers to the psychological mechanisms underlying CI. One of these mechanisms is restructuring (Ohlsson, 1984; Luo and Knoblich, 2007; Weisberg, 2015). According to representational change theory, CI is achieved by restructuring because of the biased initial problem representation. Consequently, increasing studies demonstrate the importance of restructuring in insight. For example, Öllinger et al. (2014) adopted three versions of the nine-dot problem that can evoke different representations by removing particular sources of difficulty from the standard problem, which demonstrated that three experimental versions of the problem resulted in a higher solution rate than did the standard problem. Representational restructuring/change remains necessary even when the “insight problem” doesn't involve any spatial structure change, such as brainteaser (Luo and Niki, 2003) or riddles (Fleck and Braun, 2015). Durso et al. (1994) examined restructuring in CI by having participants attempt to solve a riddle that involves a missing piece of information and observed that less misdirected representation easily resulted in CI by shortening the distance between the connections critical to restructuring. Additionally, studies on representational change training also support this role of representational change or restructuring. Patrick and Ahmed (2014), for example, documented that solution rates improved substantially with restructuring training for three different categories of insight problems, and their facilitation rates were also similar. Other mental mechanisms were also reported to manage insight (see Dietrich and Kanso, 2010); however, these mechanisms are all manifestations of the mind in CI regardless of whether the specific mechanism is representation restructuring.

Early psychologists separated the neural machinery responsible for the mind—the brain (the head)—from the body in the question of mind-body. In psychology, the mind and the brain are two independent variables; however, the mind is a complex phenomenon built on the physical scaffolding of the brain (Libet, 2006), which is a complex, temporally and spatially multi-scale structure that engenders molecular, cellular, and neuronal phenomena that together constitute the neurobiological basis of the mind (Bassett and Gazzaniga, 2011). Consequently, the term *brain* in the TIBMB primarily refers to the brain mechanisms of CI. In the past 20s years, the association between the brain and the mind has been greatly expanded. Many studies indicate that CI is related to the activations in a network of brain regions such as the bilateral prefrontal cortex, the anterior cingulated cortex, the bilateral temporal cortices, the precuneus, the insula, the amygdala, the medial temporal lobe, and the cerebellum (Shen et al., 2013). Although the precise roles of those brain regions in insight have not been entirely determined, substantial evidence across various levels reveals the complexity of insight. These levels include brain lesion investigations and neuroscience studies on healthy subjects using electrophysiological/neuroimaging (see Dietrich and Kanso, 2010) or brain stimulation techniques (Cerruti and Schlaug, 2009; Wei et al., 2014). All of the studies have documented the robust activations in those regions of insight across various tasks, indicating that CI recruits a distributed network encompassing many distant but highly connected brain regions rather than one localized area or a few specific brain regions.

Another entity that may be related to the mind in the question of mind-body is the body “below the head” (hereinafter, the term *body* refers to the body below the head), which in the human species generally comprises a neck, trunk, arms and hands, legs and feet. Beginning in the late 1980s, however, some scientists challenged the view that the body is merely an input-output facility for the brain (Libet, 2006). These scientists argued that instead, higher mental processes are grounded in bodily experience and in the neural systems that govern the body (Carpenter, 2011). The likely link between the body and CI was not recognized until recently, with mounting evidence (Grant and Spivey, 2003; Werner and Raab, 2014) that reveals the embodiment of CI. By contrast to the view of the body as an anchor or understructure of the head, increasingly more studies (Lipnicki and Byrne, 2005; Thomas and Lleras, 2007, 2009a,b) have recently demonstrated that the body may play an equal role, if not a more important role, than the brain in CI. Accumulating evidence indicates that gesturing can hinder or advance creative cognition (Garber and Goldin-Meadow, 2002), with several findings that reliably document the influence of body posture (Friedman and Förster, 2000), gestures (Lipnicki and Byrne, 2005), or bodily movement (Thomas and Lleras, 2009a) on CI. Another line of evidence for this role of body in CI is that attention guiding (Grant and Spivey, 2003; Thomas and Lleras, 2007, 2009b) or flexible eye shifting (Fleck and Braun, 2015) can facilitate insight. One common method of manipulating attention is requiring participants to perform a secondary task that can guide attentions or elicit various attention patterns by presenting visual stimuli of this secondary task in different display positions (Thomas and Lleras, 2009b). Furthermore, the automatic system acts on cardiac muscles and glands, carrying afferent signals from the vegetative organs to the brain and spinal cord, which regulate what are loosely called the body's “innards.” Kinds of critical functions like breathing are all controlled by this system (Başar, 2008). This effect of the body on CI has been replicated by studies (e.g., Jausovec and Bakracevic, 1995; Whitehurst et al., 2016) using physiological measurements on skin conduction or cardiovascular responses like heart rate variability. Overall, these lines of evidence indicate the involvement of the body in insight.

Rather than the unidirectional view that the bodily experience as a cause or a result of the insight, it seems more appropriate to take the cycled view that the insight and bodily experience both can be a cause or result of each other. If the bodily response seems to be organized and regular, or in a unchanging status that can promote self-reflective thought, this response is likely an antecedent of the insight; By contrast, if the bodily experience is less organized or only peripheroneural responses like cardiovascular activity, such experience may be a consequence of the insight. Specifically, the solvers' bodily experience of a swinging is likely an antecedent of an insight to the two-string problem (e.g., Thomas and Lleras, 2009a), which may lead to new bodily responses as a result of this insight, e.g., an increasing heart rate accompanying insight solutions (Jausovec and Bakracevic, 1995). This cycled view stresses the importance of pre-insight bodily experience/status in the insight.

Although the three key elements in the TIBMB have been discussed separately, these elements are not independent influences and generally function as an interactive system because cognitive processes are grounded in bodily experience and in the central neural systems that govern the body (Carpenter, 2011). One example of the interaction of mind and body is that individuals use their bodies to think and that cognitive processes can also result from the manner in which our bodies interact with our immediate surroundings and how “directed actions can guide thought” (Thomas and Lleras, 2009a,b). In addition, some evolutionary evidence implies the interaction of mind and body (Carpenter, 2011). In fact, the brain can be understood as a complex system that is responsible for processes or that modulates the functions of the body. The brain also modulates the cardiac muscles and glands that carry signals from the vegetative organs to the brain and spinal cord (Başar, 2008), in which mental states emerge from interactions on multiple physical and functional levels (Bassett and Gazzaniga, 2011). Meditation, for example, is known to be one method of integrative body-mind training that can improve CI performance (Ding et al., 2015) and that can be linked to the interaction of the central and autonomic nervous systems (Tang et al., 2009), and activity in the default mode network and brain connectivity (Brewer et al., 2011), supporting the TIBMB for CI. Additional support comes from Shen (2014), in which the insight experience, known to be one defining characteristic of CI (Shen et al., 2016), was revealed to be associated with the triadic interaction of brain-mind-body.

Importantly, the bodily experience should not be only regarded as a proxy of the brain. Although the bodily experience seems difficult to be entirely free of the brain due to the function of central nervous system, reliable evidence was documented that not all bodily responses were generated by these neural activities. In some situations, the bodily experience can still appear even if its corresponding central nervous system is inactive or damaged (see Bechara and Damasio, 2005), implicating the necessity of placing the body in the same position of the brain within the TIBMB. Given that similar interaction frameworks or the brain-mind-body agents might involve in some non-creative/non-insightful cognition, the specificity of the triadic interaction of them in CI should be addressed, which remains open as this field is emerging. Nonetheless, not all cognition involves the body (Irwin, 2000) and the brain and mind agents of the TIBMB are specific to CI, referring to the insight-related brain or psychological mechanisms rather than the brain or mind itself in a broader sense, suggesting the agents are specific to CI.

## The necessity of separating the brain from the entire body

In the traditional view of the mind-body relation, the brain is not separated from but is an essential component of the body (Skinner, 1990) because the brain is incorporated into the body and is essential for the consciousness or the mind. However, mounting evidence has revealed that the brain is the most important substrate of psychological processes. The special significance of the brain renders it necessary for the brain to isolate itself from the entire body, resulting in “the head” and “the body below the head.” The brain-body separation not only stresses the importance of the brain in mind or consciousness but is also helpful in illustrating the relation between the central and automatic nervous systems and their specific functions in psychological processes and some physiological phenomena. This isolation is conducive to determining precise neural correlates of types of psychological processes and facilitating CI in a variety of situations and relevant health promotion programs. With increasing interest in embodied cognition, such separation can benefit identifying the role of body components in different psychobiological/biopsychological activities and also manifest the zeitgeist of the embodied approach. Additionally, some neuroscientists have planned to conduct human head transplant experiments involving the brain-body separation (Osborne, 2016). From this perspective, the TIBMB can be considered as a theoretical response to such separation and may offer some implications into one's post-surgery behavioral/psychological change.

Collectively, we believe that the TIBMB likely play critical roles in triggering CI and studying insight experience is a key avenue validating the framework TIBMB in insight.

## Author contributions

WS and YY designed the study and wrote the manuscript. CL and JL provided intellectual input and participated in the discussion. JL, YY, and CL critically revised the manuscript. All authors have read and approved the final manuscript.

### Conflict of interest statement

The authors declare that the research was conducted in the absence of any commercial or financial relationships that could be construed as a potential conflict of interest. The reviewer FL and the handling Editor declared their shared affiliation, and the handling Editor states that the process nevertheless met the standards of a fair and objective review.
